# Children From the Age of Three Show a Developmental Switch in T-Cell Differentiation

**DOI:** 10.3389/fimmu.2020.01640

**Published:** 2020-07-28

**Authors:** Julienne Knolle, Mandy Pierau, Katrin Hebel, Karen Lampe, Gerhard Jorch, Siegfried Kropf, Christoph Arens, Monika C. Brunner-Weinzierl

**Affiliations:** ^1^Department of Pediatrics, Otto-von-Guericke-University, Magdeburg, Germany; ^2^Health Campus Immunology, Infectiology and Inflammation, Otto-von-Guericke-University, Magdeburg, Germany; ^3^Department of Otorhinolaryngology, Head and Neck Surgery, Otto-von-Guericke-University, Magdeburg, Germany; ^4^Department of Biometry and Medical Informatics, Otto-von-Guericke-University, Magdeburg, Germany

**Keywords:** infant, children, pediatric immunology, development, multifunctional T-cells, immune system, T-cell differentiation, adenoid

## Abstract

Every sixth child suffers from hypertrophy of the adenoid, a secondary lymphoid organ, at least once in childhood. Little is known about the impact of pathogen-provocation vs. developmental impact on T-cell responses after 1 year of age. Therefore, developmental and infection-driven influences on the formation of T-cell-compartments and -multifunctionality in adenoids were analyzed taking into account patient's history of age and inflammatory processes. Here, we show that in adenoids of 102 infants and children similar frequencies of naïve, effector, and memory T-cells were accumulated, whereby history of suffering from subsequent infection symptoms resulted in lower frequencies of CD4^+^ and CD8^+^ T-cells co-expressing several cytokines. While patients suffering from sole nasal obstruction had balanced Th1- and Th17-compartments, Th1 dominated in patients with concomitant upper airway infections. In addition, analysis of cytokine co-expressing CD4^+^ and CD8^+^ T-cells showed that children at the age of three or older differed significantly from those being 1- or 2-years old, implicating a developmental switch in T-cell differentiation at that age. Yet, dissecting age and infectious history of the patients revealed that while CD8^+^ T-cell differentiation seems to be triggered by development, CD4^+^ T-cell functionality is partly impaired by infections. However, this functionality recovers by the age of 3 years. Thus, 3 years of age seems to be a critical period in an infant's life to develop robust T-cell compartments of higher quality. These findings identify important areas for future research and distinguish an age period in early childhood when to consider adjusting the choice of treatment of infections.

## Introduction

Secondary lymphoid organs such as lymph nodes and spleen are important locations for the initiation of T-cell responses against pathogens. Mucosal associated lymphoid tissues that include adenoids are also secondary lymphoid organs and are confronted continuously with pathogenic antigens and function as platforms to generate adaptive immune responses for defense (1, 2). Hypertrophy of the adenoids is common in infants and younger children and predisposes to recurrent infections of the upper airways or otitis media with effusion and impaired hearing (3). Adenoidectomy at an early age often reduces these recurrent infections and restores proper hearing. Yet, even without surgery, children in some cases outgrow the chronic infections and the enlarged adenoids regress spontaneously (3–5). It is well-known that the adaptive immune response of adults and infants differs profoundly, but rather little is known about the impact of pathogen-provocation vs. developmental impact on T-cell responses after 1 year of age (6). T-cell differentiation can be understood as the delineation of T-cells into unique and defined sub-populations with certain functions. Sub-populations of CD4^+^ T-cells, namely Th1, Th2, Th9, Th17, or of CD8^+^ T-cells, Tc1, Tc2, and Tc17, are mainly defined by their cytokine production (1, 7). For neonates and infants, a Th2 bias has been suggested, but was not visible upon inflammation and pathogen confrontation (2, 8, 9). In addition, fetal and neonatal T-cells express enhanced the signatory cytokines IL-8 or IL-17, yet also IL-10 has been reported (8, 10, 11). That this signatory cytokine production is not as straight forward in infants and children, as no enhanced IL-8 or IL-17 expression was detectable in a cohort of 1 to 5 year old children at different mucosal sites (12). Although Th17 cells are discussed to be essential in children for mucocutaneous immunity especially against *Candida* and *Staphylococcus* in the respiratory tract and on the skin, and Th1 cells for protection against intracellular antigens, a balance of both seems to be optimal (13). In terms of CD8^+^ T-cell responses, they are suggested to not be generated properly before the age of two (14, 15). Nevertheless, other associated studies suggest that infections, treatment with antibiotics as well as individual variations in immune development during childhood have long-term consequences to the prevalence of many diseases (16–18). However, detailed knowledge of molecular and cellular mechanisms is missing to fully understand T-cell responses early in life.

A strategy for the evaluation of CD4^+^ and CD8^+^ T-cell functionality is based on the co-expression of different cytokines by individual T-cells, which provides them with different effector functions as well as cytokine-mediated expansion (19, 20). In general, these multifunctional T-cells are strongly associated with protection against pathogen-threat and fast clearance of infections. Consequently, vaccines are thought to improve the protection against infections mainly through the formation of multifunctional T-cells (21–23). However, the capability of infants to form substantial amounts of multifunctional T-cells is still not clear.

In the present study, adenoids were chosen to analyze the formation of cytokine co-expressing T-cells, which served as an indication for the ability of the organism to generate complex adaptive immune responses. Circumstances of acute and chronic infections as possible driving forces for the formation of T-cell differentiation were compared with developmental changes with age within the general development of children's immune system.

## Methods

### Study Design

The study was approved by the Clinical Research Ethics Board of the University of Magdeburg (N° 06/11) and all patient parents provided written informed consent at the time of enrollment in accordance with the Declaration of Helsinki. One hundred and two children aged between 1 and 11 years suffering from adenoid hypertrophy who underwent adenoidectomy at the Department of Otorhinolaryngology, Head & Neck Surgery at the University Hospital of Magdeburg were included in the study. Children suffering from immune or genetic diseases were excluded. According to predominant clinical symptoms of the children at the time of surgery (Table 1), three groups were compared, two infection groups with either recurrent infections of the upper airways or concomitant otitis media with effusion and one none-infection group presenting with nasal obstruction only.

**Table 1:** Descriptive statistics of patients included in the study.

**Clinical group**	**Symptoms**		**Sex**		**Total patients**
			**M**	**F**	
**(A)**					
Group 1 Nasal obstruction	Clinical Nasal obstruction	Count	19	10	29
		% within Group 1	100.0%	90.9%	96.7%
	Snoring	Count	17	8	25
		% within Group 1	89.5%	72.7%	83.3%
	“Nasal” pronounciation	Count	11	3	14
		% within Group 1	57.9%	27.3%	46.7%
	***Total Group 1***	***Count***	***19***	***11***	***30***
		***% within Group 1***	***63.3%***	***36.7%***	***100.0%***
Group 2 Upper airway infections	Recurrent rhinitis/pharyngitis	Count	14	19	33
		% within Group 2	100.0%	100.0%	100.0%
	Recurrent bronchitis	Count	8	13	21
		% within Group 2	57.1%	68.4%	63.6%
	***Total Group 2***	***Count***	***14***	***19***	***33***
		***% within Group 2***	***42.4%***	***57.6%***	***100.0%***
Group 3 Otitis media with effusion	Recurrent otitis	Count	12	8	20
		% within Group 3	50.0%	53.3%	51.3%
	Hearing impairment	Count	23	13	36
		% within Group 3	95.8%	86.7%	92.3%
	Delay in speech development	Count	17	12	29
		% within Group 3	70.8%	80.0%	74.4%
	***Total Group 3***	***Count***	***24***	***15***	***39***
		***% within Group 3***	***61.5%***	***38.5%***	***100.0%***
	**Total**	**Count**	**57**	**45**	**102**
		**% within all Patients**	**55.9%**	**44.1%**	**100.0%**
**Age**		**Sex**			**Total patients**
		**M**	**F**		
**(B)**					
1 year	Count	17	8		25
	% within Age	68.0%	32.0%		100.0%
2 years	Count	16	16		32
	% within Age	50.0%	50.0%		100.0%
≥3 years	Count	24	21		45
	% within Age	53.3%	46.7%		100.0%
**Total**	**Count**	**57**	**45**		**102**
	**% within Age**	**55.9%**	**44.1%**		**100.0%**

*(A) 102 patients were organized in three clinical groups according to predominant symptoms and disclosing gender. Group 1 contains children suffering from sole nasal obstruction, whereas in the other two groups children also show concomitant infections of the upper airways (group 2) or of the middle ear leading to effusion, hearing impairment and sometimes even impairment in speech development (group 3). For each group, the children's medical history is specified by the clinical symptoms they had presented with in the past. The size of each group in respect to the entire collective is indicated as percentage. (B) All patients were grouped according to age at time of surgery and disclosing gender. Children between the age of 1 and 11 years were included in the study. Comparisons were made between children of 1, 2, and 3 years and older. The size of each group in respect to the entire collective is indicated as percentage. All 102 patients could be analyzed*.

### Analysis of Immune Competence

Mononuclear cell (MC) suspensions were generated from adenoids in PBS-BSA by mechanical dissociation over a 70-μm cell strainer (BD Biosciences, San Jose, CA) (2). 1–5 x 10^7^ MCs were cryopreserved at −80°C in 1 ml DMSO and FCS in a ratio 1:10. For cell culture MCs were thawed, washed, resuspended in x-vivo-15 media (Lonza, Belgium) and rested overnight at 37°C. 5 x 10^6^ MCs/ml were stimulated with 10 ng/ml PMA and 1 μg/ml Ionomycin in the presence of 5 μg/ml Brefeldin A (Sigma-Aldrich, St. Louis, MO) for 6 hr. Unstimulated cells were used for phenotyping of CD4^+^ and CD8^+^ T-cell subsets. For intracellular analysis, cells were fixed in 2% paraformaldehyde (Morphisto) and permeabilized with 0.5% saponin (Sigma) (24). Cytometric data were acquired on a FACS-Canto II (BD) and analyzed with FlowJo (FlowJo LLC) software. The following fluorochrome-conjugated Abs were used for flow cytometry: anti-CD3 (SK7), anti-CD45 (H130), anti-CD4 (RPA-T4), anti-CD8 (RPA-T8), anti-CD31, anti-CD45RO (UCHL1), anti-CD45RA, and anti-CD27, anti-IL-2 (MQ1-17H12), anti-TNFα (Mab11), anti-IL-17 (eBio64DEC17) and anti-IFNγ. The gating strategy for cytokine expression and phenotyping of CD4^+^ and CD8^+^ T-cell subsets are shown in the supplemental (Figures S1, S2). T-cell subpopulations showing single, double, triple and quadruple cytokine producers were analyzed by applying Boolean gating on either CD3/CD4 or CD3/CD8 T-cells.

### Statistical Analysis

Statistical analyzes were performed using GraphPad Prism6 and SPSS22. Normal distributions of variables were checked with a Kolmogorov-Smirnoff test. For pairwise comparisons, either Student *t-*test or non-parametric Mann-Whitney test was used depending on Gaussian distribution. For multiple comparisons, Kruskal-Wallis method with *post-hoc* Dunn multiple comparison or one-way ANOVA with a Bonferroni test were used. Furthermore, two-way-ANOVA multiple comparisons in conjunction with a Tukey HSD (honest-significance-difference) test or Spearman correlations were performed as indicated. Statistical significance was considered for *p* < 0.05. ^*^p < 0.05, ^**^p < 0.01, ^***^p < 0.001, ^****^p < 0.0001. Results are expressed as mean ± standard error of mean (SEM).

## Results

### Substantial Amounts of Effector and Memory T-Cells Persist in Pediatric Adenoids

**Figure 1:**
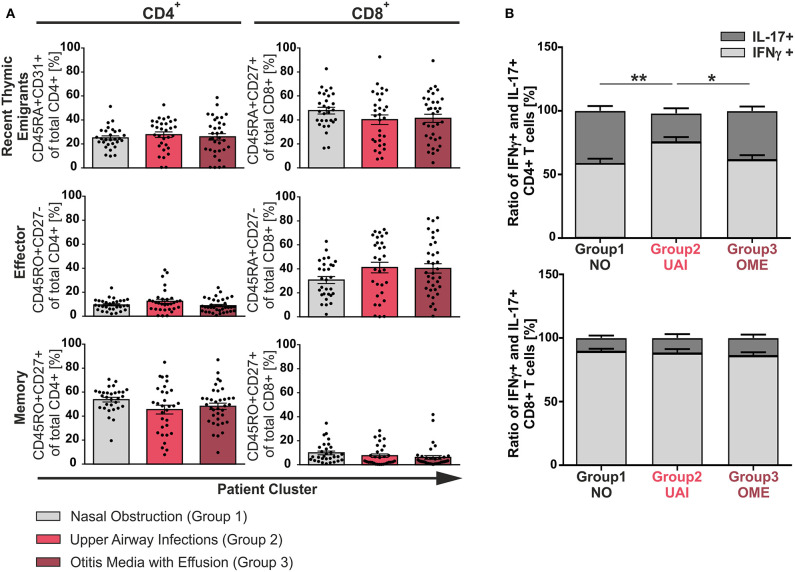
Phenotype of CD4^+^ and CD8^+^ T-cells among the three clinical groups. Mononuclear cells (MC) were isolated from adenoids of 102 children suffering from adenoid hypertrophy that underwent adenoidectomy. MCs of children with sole nasal obstruction (group 1), with those suffering from recurrent infections of the upper airways (group 2), or with concomitant otitis media with effusion (group 3) were either left unstimulated for phenotyping or stimulated for analysis of cytokine production with PMA and Ionomycin in the presence of Brefeldin A for 6 h. Surface marker or cytokine expression of CD4^+^ and CD8^+^ T-cells was determined by using flow cytometry. **(A)** Frequencies of naive/recent thymic emigrants, effector, and memory T-cells were compared depending on predominant clinical symptoms, each shown for total CD4^+^ (left side) and CD8^+^ (right side) T-cells. Children suffering from sole nasal obstruction (group 1, light gray bars), suffering from recurrent upper airway obstructions (group 2, red bars), or suffering from concomitant otitis media with effusion (group 3, dark red bars) are depicted. Each dot represents an individual patient out of *n* = 102. Horizontal lines indicate mean ± SEM (one-way ANOVA, Tukey HSD multiple comparisons). **(B)** The percentages of total IFNγ^+^ (light gray) or IL-17^+^ (dark gray) CD4^+^ (upper panel) and CD8^+^ (lower panel) T-cells were determined, calculated to hundred percent, and presented in a comparative manner. Kolmogorov-Smirnoff test as well as a Kruskal-Wallis method with *post-hoc* Dunn multiple comparison were used. Horizontal lines indicate mean ± SEM. *p < 0.05, **p < 0.01.

**Figure 2:**
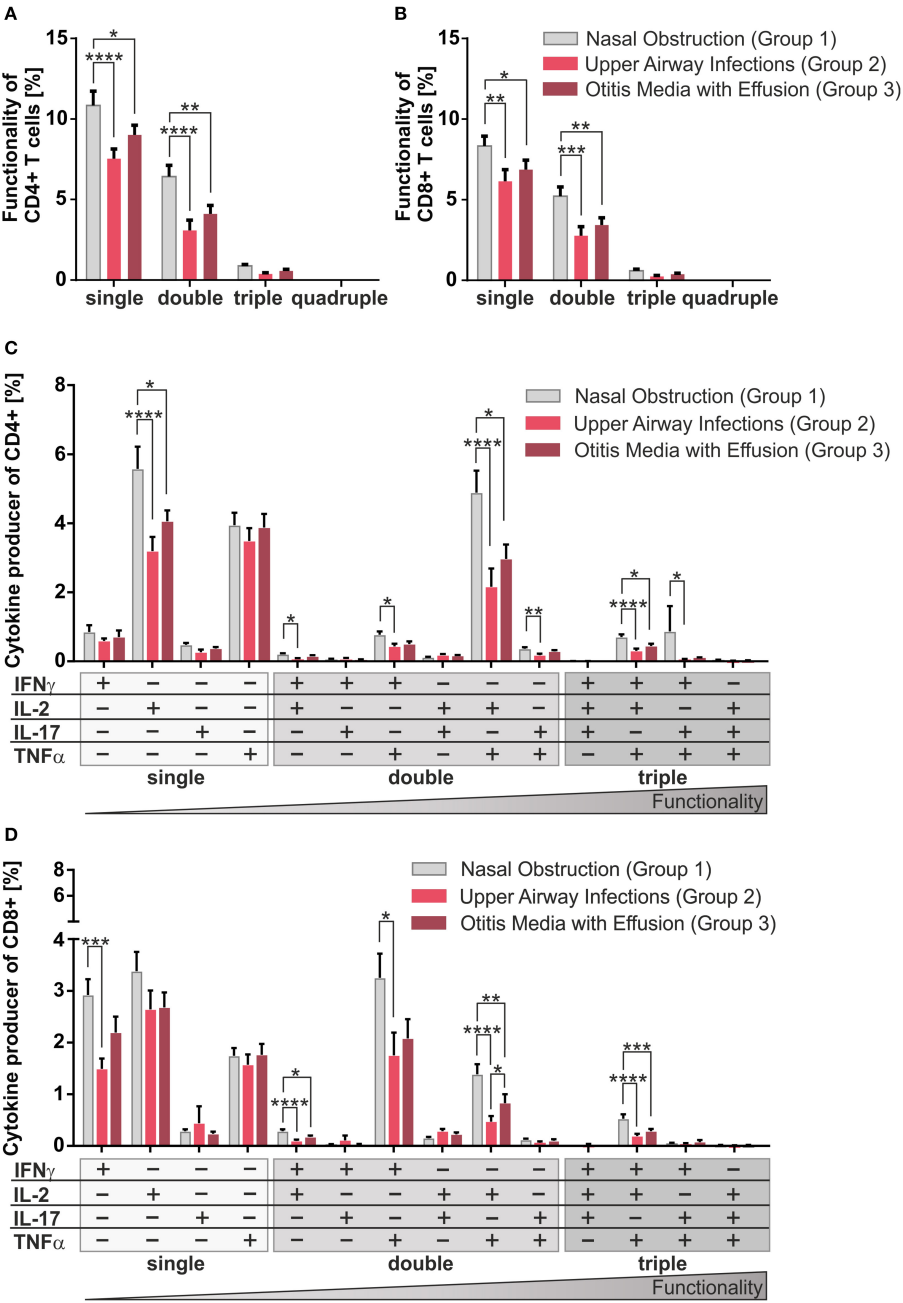
Multifunctionality of CD4^+^ and CD8^+^ T-cells among the three clinical groups. MCs of pediatric adenoids were stimulated as described in Figure 1. Intracellular cytokine expression of CD4^+^ and CD8^+^ T-cells was determined by using flow cytometry and the Boolean gating strategy. **(A,B)** Frequency of summarized CD4^+^ and CD8^+^ subpopulations (summarized subsets that express just one (single), or simultaneously two (double), three (triple), or four (quadruple) different cytokines) among all CD4^+^ **(A)** and CD8^+^ **(B)** T-cells. **(C,D)** Frequencies of individual CD4^+^ and CD8^+^ subpopulations (+ indicates the expressed cytokine) among all CD4^+^ **(C)** and CD8^+^ **(D)** T-cells. Light gray bars represent children suffering from sole upper airway obstruction (group 1), whereas red bars represent children additionally suffering from recurrent upper airway infections (group 2), and dark red bars stand for children with concomitant otitis media with effusion (group 3). The triangle symbolizes that the functionality of the T-cells increases with the number of cytokines produced simultaneously (two-way ANOVA, Tukey HSD multiple comparisons) *p < 0.05, **p < 0.01, ***p < 0.001, ****p < 0.0001.

To identify characteristics of T-cell differentiation, a cohort of 102 pediatric patients aged 1–11 years undergoing adenoidectomy because of hypertrophy were analyzed as a model for inflammation-driven or developmental driven forces on T-cell functionality. Clinical history of patients was exploited to be able to group patients according to previous suffering from acute or chronic infections, namely concomitant otitis media with effusion (group 3, OME) or recurrent infections of the upper airways (group 2, UAI). Another group suffering only from nasal obstruction was considered to represent the non-infectious group (group 1, NO). An *ex vivo* analysis of single cell suspensions of adenoids was performed using flow cytometric immunotyping of CD4^+^ and CD8^+^ T-cells (Figures 1, 2, and Figures S1, S2). Accumulation of recent thymic emigrants (RTE), considered as CD4^+^CD45RA^+^CD31^+^ and CD8^+^CD45RA^+^CD27^+^ T-cells, were similar in the three patient groups, and thus identified to be independent of infection history (Figure 1A). In terms of antigen-experienced T-cells, similar frequencies of effector (CD4^+^CD45RO^+^CD27^−^ and CD8^+^CD45RA^+^CD27^−^) and memory (CD4^+^CD45RO^+^CD27^+^ or CD8^+^CD45RO^+^CD27^+^) T-cells were observed within the three disease groups suggesting that the size of these compartments is independent of inflammation caused by infections. Surprisingly, even in the non-inflammatory group 1, infants and children showed similar frequencies of memory T-cell populations as patients with infections (Figure 1A) (14). Of note, the naïve and effector CD8^+^ T-cell compartments of all adenoids analyzed were much larger than the one of CD4^+^ T-cells (Figure 1A). However, the CD4^+^ T-cell population was comprised of a notably larger memory compartment than that of the CD8^+^ one (Figure 1A).

### Reduced Frequencies of Multifunctional T-Cells in Children With Infectious Complications

Having found the size of CD4^+^ and CD8^+^ compartments of memory and effector T-cells to be independent of infection history, differentiation was monitored in our model using cytokine production as surrogate marker (Figures 1B, 2). Even though there was a balance of IFNγ and IL-17 producers in the CD4^+^ compartment as needed to effectively fight infections (Figure 1B, upper panel), frequencies of Th17 cells clearly dropped in half by patients suffering from upper airway inflammations (group 2). In the CD8^+^ compartment Tc1 cells dominated independent of infections (Figure 1B, lower panel).

Single, double, and triple cytokine producers of CD4^+^ and CD8^+^ T-cells were monitored in all disease groups (Figures 2A,B). Single and double producers dominated the response in both compartments and were significantly increased in children with sole nasal obstruction (group 1) compared to the other two groups; quadruple producers were hardly visible. As triple producers of TNFα, IL-2, plus IFNγ are known to be strongly associated with pathogen clearance (21), special attention was payed to them by dissecting triple producers into individual functional T-cell subsets (Figures 2C,D). Children with sole nasal obstruction (group 1) showed significantly more of those Th1-like and Tc1-like triple producers than those in the two infection-biased clinical groups (Figures 2C,D). As Th17 and Tc17 cells are known to show high plasticity to acquire the ability to co-express the Th1-lineage cytokine IFNγ (25), IL-17 producing triple producers were also monitored. Along with the before-mentioned triple-producers, the IFNγ/IL-17 co-expressing population was mainly detected together with TNFα co-expression in CD4^+^ T-cells of patients without infectious history (Figure 2C).

Overall, similarly for the CD4^+^ (Figures 2A,C) and the CD8^+^ compartments (Figures 2B,D), patients from the non-infectious group 1 differed profoundly from the other two groups, showing enhanced frequencies of co-producers, thus functionally better equipped T-cells (21).

### Effector and Memory T-Cell Compartments Are Overall Similar in Adenoids of Infants and Children

**Figure 3:**
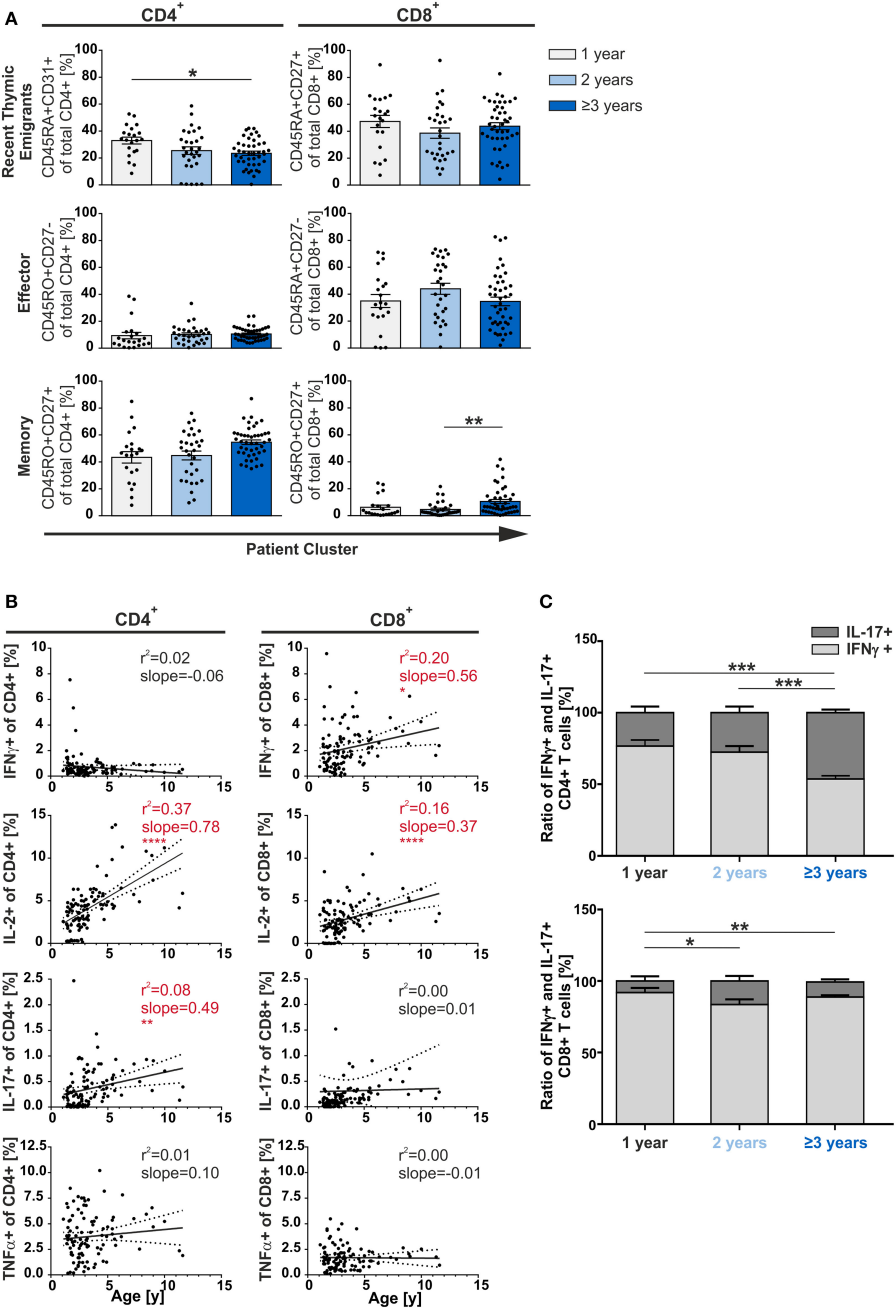
Phenotype of CD4^+^ and CD8^+^ T-cells depending on age. MCs of adenoids of children being 1, 2, or 3 years and older with adenoidectomy were stimulated and analyzed as in Figure 1. **(A)** Frequencies of naive/recent thymic emigrants, effector, and memory T-cells are compared depending on age at the time of surgery, each shown for total CD4^+^ (left side) and CD8^+^ (right side) T-cells. One-year-old (light gray bars), 2-year-old (light blue bars), and 3-year-old and older (dark blue bars) infants and children are depicted. Each dot represents an individual patient from *n* = 102 infants and children included in the study. Horizontal lines indicate mean ± SEM (one-way ANOVA, Tukey HSD multiple comparison). *p < 0.05, **p < 0.01. **(B)** Line graphs represent linear regression with 95% confidence intervals among CD4^+^ and CD8^+^ T-cells. Functional populations for single producers for each individual cytokine analyzed within participants by advancing age (1–11 years). Line graph statistics were analyzed by Spearman *r* correlation (*p < 0.05, **p < 0.01, ****p < 0.0001). **(C)** The percentages of total IFNγ^+^ (light gray) or IL-17^+^ (dark gray) CD4^+^ (upper panel) and CD8^+^ (lower panel) T-cells were determined, calculated to hundred percent, and presented in a comparative manner. Kolmogorov-Smirnoff test as well as a Kruskal-Wallis method with *post-hoc* Dunn multiple comparison were used. Horizontal lines indicate mean ± SEM. *p < 0.05, **p < 0.01, ***p < 0.001.

As multiple infections were not leading to enhanced differentiation/accumulation of multifunctional T-cells (Figure 2), we asked whether the age of the patients could actually be decisive. Therefore, the patients were grouped by age and the T-cell compartments were then analyzed accordingly. Results demonstrated that frequencies of CD8^+^ RTE and effector T-cells did not show any differences between age groups. In the CD4^+^ compartment, only the frequency of RTE but neither of effector nor of memory cells showed a significant dependency on age (Figure 3A). This also demonstrates that already 1 year old infants generate substantial amounts of memory CD4^+^ T-cells. When comparing children of 3 years and older with 2-year olds, the former were seen to have about twice as many CD8^+^ memory T-cells although at very low frequencies (*p* < 0.01) (Figure 3A).

Next, we analyzed the single cytokine producing cells in terms of aging during early childhood. Among all CD4^+^ and CD8^+^ T-cells, single-producers increase steadily and significantly (slope_CD4_ 1.44; slope_CD8_ 0.56; data not shown). This increase was mainly due to the CD4^+^ IL-2 and IL-17 and the CD8^+^ IFNγ and IL-2 single producers (Figure 3B). Besides age differences, CD4^+^ single TNFα and single IFNγ producers as well as CD8^+^ single IL-17 and single TNFα producers were generated at equal frequencies.

As IL-17 was described as a signatory cytokine in fetal and neonatal T-cells (26), we further focused on Th17 cells. Unexpectedly, Th1-like cells were found to be more abundant than Th17-like cells in younger infants (1- and 2-year-olds) while frequencies of Th17 cells significantly rose with age in adenoids of 1 to 11-year olds (Figures 3B,C). Indeed, older children (≥3 years) showed 50% (*p* < 0.001) more IL-17 producing CD4^+^ T-cells than those being 1 or 2-years old, leading to a Th1:Th17 balance in the CD4^+^ compartment (Figure 3C). The CD8^+^ compartment was always dominated by Tc1 when compared to Tc17 cells, with a significant doubling of the frequency of Tc17 cells from the age of 2 years—albeit in low frequency (Figure 3C).

### Frequencies of Multifunctional CD4^+^ and CD8^+^ T-Cells Are Increased From the Age of 3 Years Onward

**Figure 4:**
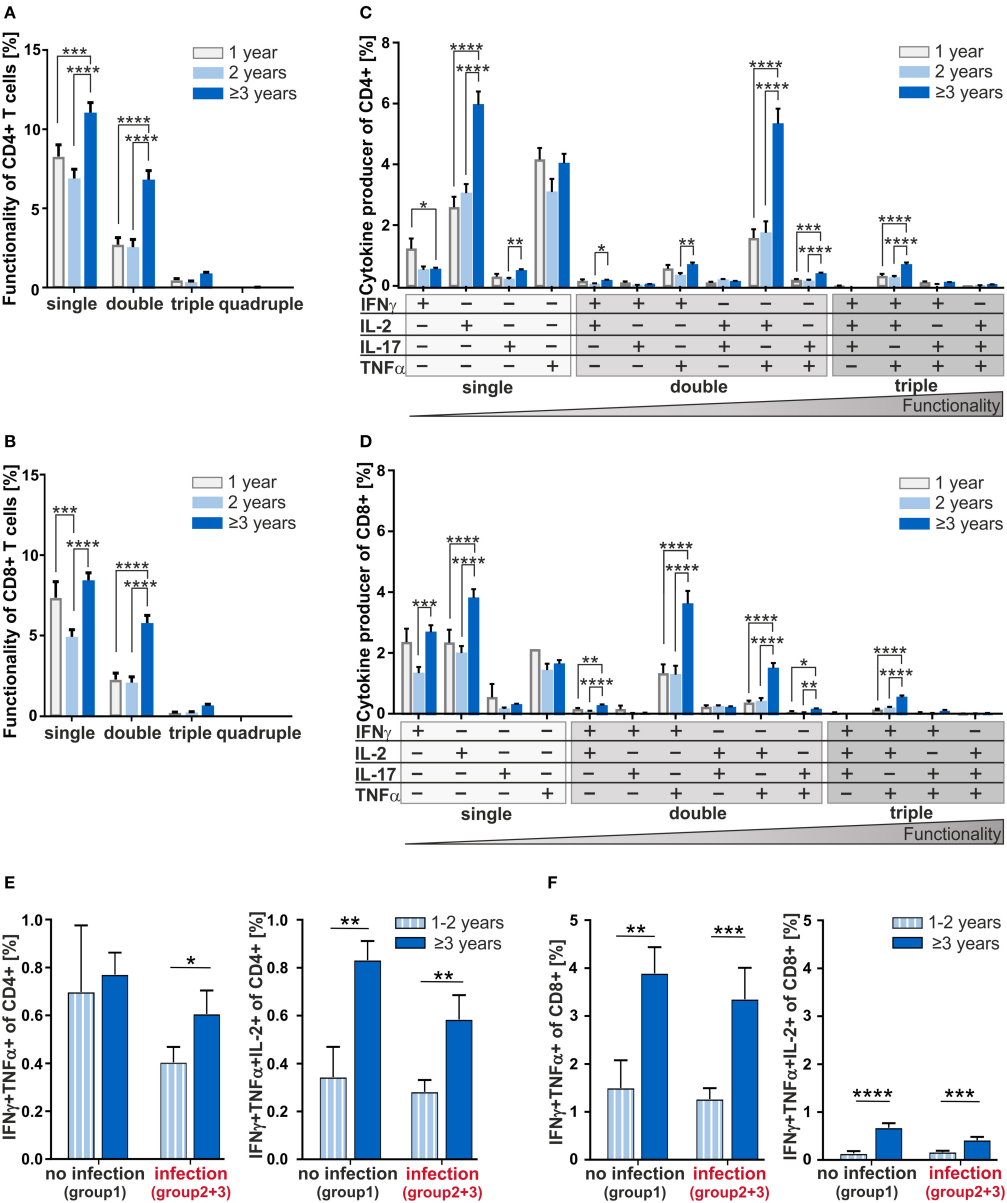
Multifunctionality of CD4^+^ and CD8^+^ T-cells depending on age and cytokine co-expression profiles depending on the infection status. MCs of adenoids from children being 1, 2, or 3 years and older with adenoidectomy were stimulated as in Figure 1 and analyzed as in Figure 2. **(A,B)** Frequencies of summarized CD4^+^ and CD8^+^ subpopulations (summarized subsets that express just one (single), or simultaneously two (double), three (triple), or four (quadruple) different cytokines) among all CD4^+^ **(A)** and CD8^+^ **(B)** T-cells. **(C,D)** Frequencies of individual CD4^+^ and CD8^+^ subpopulations (+ indicates the expressed cytokine) among all CD4^+^ **(C)** and CD8^+^ **(D)** T-cells. Children being 1 year old (light gray bars) at the time of surgery, children being 2 years old (light blue bars), and children being 3 years and older (dark blue bars) are depicted. The triangle symbolizes that the functionality of the T-cells increases with the number of cytokines produced simultaneously (two-way ANOVA, Tukey HSD multiple comparisons). *p < 0.05, **p < 0.01, ***p < 0.001, ****p < 0.0001. **(E,F)** Frequencies of IFNγ and TNFα double positive CD4^+^ **(E)** and CD8^+^ **(F)** T-cells in children being 1 and 2 years of age or 3 years and older either with (summarized group 2 and 3) or without (group 1) infections are shown on the left, while frequencies of IFNγ, TNFα, and IL-2 triple positive CD4^+^ **(E)** and CD8^+^ **(F)** T-cells are shown on the right. White and light blue bars represent children being 1 and 2 years of age whereas dark blue bars represent children of 3 years and older at the time of surgery. Horizontal lines indicate mean ± SEM (Kolmogorov-Smirnoff test; Kruskal-Wallis method with *post-hoc* Dunn multiple comparison). *p < 0.05, **p < 0.01, ***p < 0.001, ****p < 0.0001.

Next, to investigate the age-dependency on functionality of CD4^+^ and CD8^+^ T-cells in adenoids, we analyzed individual cytokine co-expressing T-cells in 1, 2, and 3 years and older children (Figure 4). Summarizing the cytokine producers within the CD4^+^ and CD8^+^ compartments as single, double-, triple-, and quadruple-producers, respectively, a significant increase of single- and double-producers was observed in the oldest group compared to the 1- and 2-year-old infants (Figures 4A,B). These data show a clear difference becoming visible in the functionality of T-cells during and after the third year of life.

Analyzing CD4^+^ double producers, IL-2^+^TNFα^+^ co-producers accounted for the largest subpopulation and children being 3 years and older accounted for 70% more IL-2^+^TNFα^+^ co-producers than 1- and 2-year-olds (Figure 4C). Indeed, differences could be seen between children of 3 and those being 2 years of age for most of the double producers analyzed, i.e., IFNγ co-producers with IL-2^+^or TNFα^+^ as well as for IL-17^+^TNFα^+^ co-producers.

Overall, the functionality of CD4^+^ T-cells determined by co-expression of IFNγ, IL-2, IL-17, and TNFα increased significantly with age (Figures 4A,C). For most of the individual subpopulations according to cytokine expression, functional capacity of T-cells in 1- and 2-year-old children were hardly distinguishable. The IFNγ^+^IL-2^+^TNFα^+^ co-expressing CD4^+^ and CD8^+^ T-cells which are known to correlate with pathogen clearance (21), were shown at a significantly higher frequency in children being 3 years and older compared to those of one and 2 years of age (Figures 4C,D). Concerning these triple-positive T-cells in the CD8^+^ compartment, children of at least 3 years of age accounted for 77% more (*p* < 0.0001) than children of one and 65% more (*p* < 0.0001) than children of 2 years of age (Figure 4D). Thus, 3-years- and older children who display significantly higher frequencies of double- and triple-cytokine co-producing T-cells show increased T-cell functionality.

Taken together, with increasing age, children can be described to harbor functionally more complex CD4^+^ and CD8^+^ T-cells such as double- and triple-producers. For younger children, a higher amount of functionally less equipped T-cells dominate.

### Developmental Impact on the Functionality of CD4^+^ and CD8^+^ T-Cells

At first glance, the ability to co-express cytokines simultaneously seemed to be based on a non-inflammatory and development-specific process. In order to separate the influence of development and infection on the generation of cytokine co-expressing T-cells, we next analyzed the age dependency of the generation of these co-expressing CD4^+^ and CD8^+^ T-cells taking into consideration the infection history (Figures 4E,F). In the none-infection group of infants and children, the CD4^+^IFNγ^+^TNFα^+^ double producers were identified to be similar across different ages (Figure 4E, left). However, focusing on children with infection history, 1- and 2-year-olds display dramatically reduced frequencies of these double producers. Data also show that children from the age of three show an increase in the double producers despite infections. Having a look at the CD4^+^IFNγ^+^TNFa^+^IL-2^+^ triple producers, a drastic increase with age was visible independent of infection history (Figure 4E, right). Clearly, CD8^+^IFNγ^+^TNFα^+^ double producers and albeit in lower frequencies CD8^+^IFNγ^+^TNFa^+^IL-2^+^ triple producers increased considerably with 3 years of age, regardless of any acute or chronic infection in earlier life (Figure 4F). Consequently, while infections seem to be responsible for reduced frequencies of IFNγ^+^TNFα^+^ co-expressing CD4^+^ T-cells in the first 2 years of life, an increase of triple producers is merely due to development. This contrasts with the CD8^+^ T-cells, whose increase of any kind of cytokine co-producers is determined exclusively by age—thus, purely developmental.

## Discussion

To better understand the functionality of pediatric T-cell responses, T-cell functionality was studied in 102 children suffering from adenoid hypertrophy, either in combination with acute or with chronic infections or none.

Here, we show that in a secondary lymphoid organ, the adenoid, a consistently similar frequency of naïve, effector, and memory T-cells is maintained in patients aged 1–11 years, with no general impact of infection history or age (Figures 1A, 3A). In blood, a steady increase in circulating memory T-cells and decrease in naïve T cells is discussed (6, 27, 28). However, the consistency of T-cell compartment sizes in adenoids suggests functional significance. Herein, memory CD4^+^ T-cells were the predominant population in adenoids starting at 1 year of age, followed by recent thymic emigrants, while the latter comprised the largest proportion of CD8^+^ T-cells. For other tissues and blood in infants it was reported that local T-cell compositions are mainly made up of naive T-cells (12, 29). The local accumulation of a high proportion of CD4^+^ memory T-cells especially in adenoids early in life likely reflects the requirement of immediate defense to the recurrent exposure to pathogens. The CD8^+^ compartment in the data consistently displayed low frequencies of memory cells in comparison to effector and naïve cells. This is in line with previous findings, which report that CD8^+^ T-cells do not easily generate memory and the same may apply to at least this pediatric relevant period or site (30).

Direct killing of infected cells as part of the protection against pathogens might explain the large proportion of CD8^+^ effector T-cells compared to CD8^+^ memory T-cells in the adenoids. Mechanistically, either the challenging environment or cell-intrinsic mechanisms support a preferred differentiation in effector T-cells rather than into memory T-cells. Effector T-cells are advantageous because they react faster than memory CD8^+^ T-cells (31). However, if the enhanced proportion of CD8^+^ effector T-cells in children is caused by the hypothesized layered immune system at birth, meaning that the T-cell differentiation based on different progenitor cells still exists in parallel, this needs to be further elaborated (30).

Although frequencies of effector and memory T-cells do not change dramatically in adenoids, a continuous change in the TCR repertoire can be expected, as colonization of mucosal sites increases diversity within the first 3 years of life when the phylogenetic composition of the bacterial communities evolve toward an adult-like configuration (12, 32).

Even though patients with sole nasal obstruction could be expected to suffer from infections for organic and steric reasons alone, our results would not support such a scenario. As especially those patients in general generate higher frequencies of cytokine co-producers than those in the other two groups with acute and chronic infectious complications, and they can be interpreted as having a better disease-control (Figure 2). This is in line with many studies showing that these multifunctional T-cells are needed to clear infections and are superior to single-producers (20, 33–36). Indeed, this T-cell profile of higher quality seems to be protective against infectious complications in children with enlarged adenoids (Figure 2). These results are also supported by vaccination studies showing that vaccines eliciting multifunctional CD4^+^ or CD8^+^ T-cells ensure better protection against viral infections, e.g., a significant increase in multifunctional T-cells following repeated annual influenza vaccination was recently reported (21–23). Furthermore, HPV-vaccines were illustrated to enhance the control of HPV-associated tumors by activation of multifunctional CD8^+^ T-cells (37). This further strengthens our data that multifunctionality of T-cells enhances protection also in pediatric patients and leads to a better control of pathogen-provocation.

While CD4^+^ T-cells of patients with upper airway infections showed a Th1 dominance, the group with only nasal obstruction showed an optimal Th1/Th17 ratio of one (Figure 1B). This is in agreement with the report that a balanced ratio of Th1 and Th17 cells in adenoids is advantageous (13). Nevertheless, patients with acute infection (Figure 1B, group 3), also show a balance of Th1/Th17 ratio and it is tempting to speculate that it may still be beneficial to prevent chronicity. Overall, the reported IL-17-bias of fetal and neonatal cells does not account for T-cells in early childhood (2, 38). The disappearance of the fetal/neonatal-monitored Th17-bias could be explained by the high plasticity of Th17 cells to convert to Th1-like cells (25), or by a different origin of the responding cells (30). Indeed, absence of Th17 dominance points toward a replacement of the suggested neonatal precursor cells postpartum rather than to the co-existence of a layered immune system (13).

Age strongly appears to influence the magnitude and quality of the T-cell responses and being 3 years or older seems to mark a turning point in regard to T-cell quality. Knowledge about the effect of age on multifunctionality of T-cells in secondary lymphoid organs is scarce (2) and to our knowledge, so far, no *ex vivo* data from secondary lymphoid organs are available from multifunctionality of T-cells in a pediatric cohort as young as ours. However, age effects on T-cell multifunctionality in older children (11–17 years) was suggested in a small study using circulating T-cells (34). In adulthood, frequency of cytokine co-expressing T-cells is stable (39, 40).

In the CD8^+^ compartment, the generation of multifunctionality demonstrates especially a clear-cut increase from 3 years of age with a highly significant impact of age, but not of infections (Figure 4F). However, a switch from low frequencies of multifunctional as well as memory CD8^+^ T-cells early in life could point toward missing differentiation-supporting structures within the secondary lymphoid structure. Indeed, also other subpopulations of lymphocytes that are dependent on secondary lymphoid structures such as mature marginal zone B cells, start to accumulate in substantial amounts not before 2 years of age (41, 42). Thus, even after 1 year of life, functionality analysis of T-cells in adenoids demonstrates that the development of the immune system still follows a developmental trajectory (43). Hence, the influence of developmental processes on immune functioning throughout life span could be far greater than that is apparent at first glance (44, 45).

Importantly, in comparison to the CD8^+^ compartment the CD4^+^ compartment behaves differently in the way that age and infections impact the formation of cytokine co-producers (Figure 4E). Whereas, IFNγ^+^TNFα^+^IL-2^+^ triple producers are similar in infectious and non-infectious groups before the age of three, IFNγ^+^TNFα^+^ double producers differ profoundly. This implicates that filling the compartment of triple producers during infections might happen on the expense of double producers. As these triple producers are more prone to enter the memory pool, this might be of ontogenetic significance (21). Therefore, the ability to generate more complex T-cell-responses seems to be an internal mechanism within the development of a child's immune system.

In conclusion, the comparison of these young age groups according to cytokine co-expression and clinical symptoms might help physicians designing conditioning protocols adapted to age, taking into account the better control of infections and a fully equipped immune system already after 3 years of age. These findings also outline from an immunological point of view, that it is important to consider age and acute or chronic infection history when deciding upon clinical procedures, thus, the importance of adapting indications and protocols according to expected immune fitness.

## Data Availability Statement

All datasets presented in this study are included in the article/Supplementary Material.

## Ethics Statement

The studies involving human participants were reviewed and approved by Clinical Research Ethics Board of the University of Magdeburg (No. 06/11). Written informed consent to participate in this study was provided by the participants' legal guardian/next of kin. Written informed consent was obtained from the minor(s)' legal guardian/next of kin for the publication of any potentially identifiable images or data included in this article.

## Author Contributions

MB-W and GJ designed the study. MB-W, KL, GJ, and CA participated in the interpretation of the findings, read, and critically revised the manuscript. JK contributed to the design of the work, performed experiments, interpreted the findings, and wrote the manuscript together with MB-W and MP. KH participated in data analysis and revised the manuscript. MP participated in data analysis and data interpretation and wrote the manuscript. SK has made the statistic. All co-authors read the manuscript and have provided important intellectual input to the manuscript. The corresponding author had full access to the data and had final responsibility for the decision to submit for publication.

## Conflict of Interest

The authors declare that the research was conducted in the absence of any commercial or financial relationships that could be construed as a potential conflict of interest.
