# Supplementary material for: Children From the Age of Three Show a Developmental Switch in T-Cell Differentiation
Source: Front Immunol. 2020 Jul 28;11:1640. doi: 10.3389/fimmu.2020.01640 (PMC7402172; doi:10.3389/fimmu.2020.01640)
Supplement: Supplementary file 1 [file Data_Sheet_1.pdf]

Supplemental Information

Figure S1

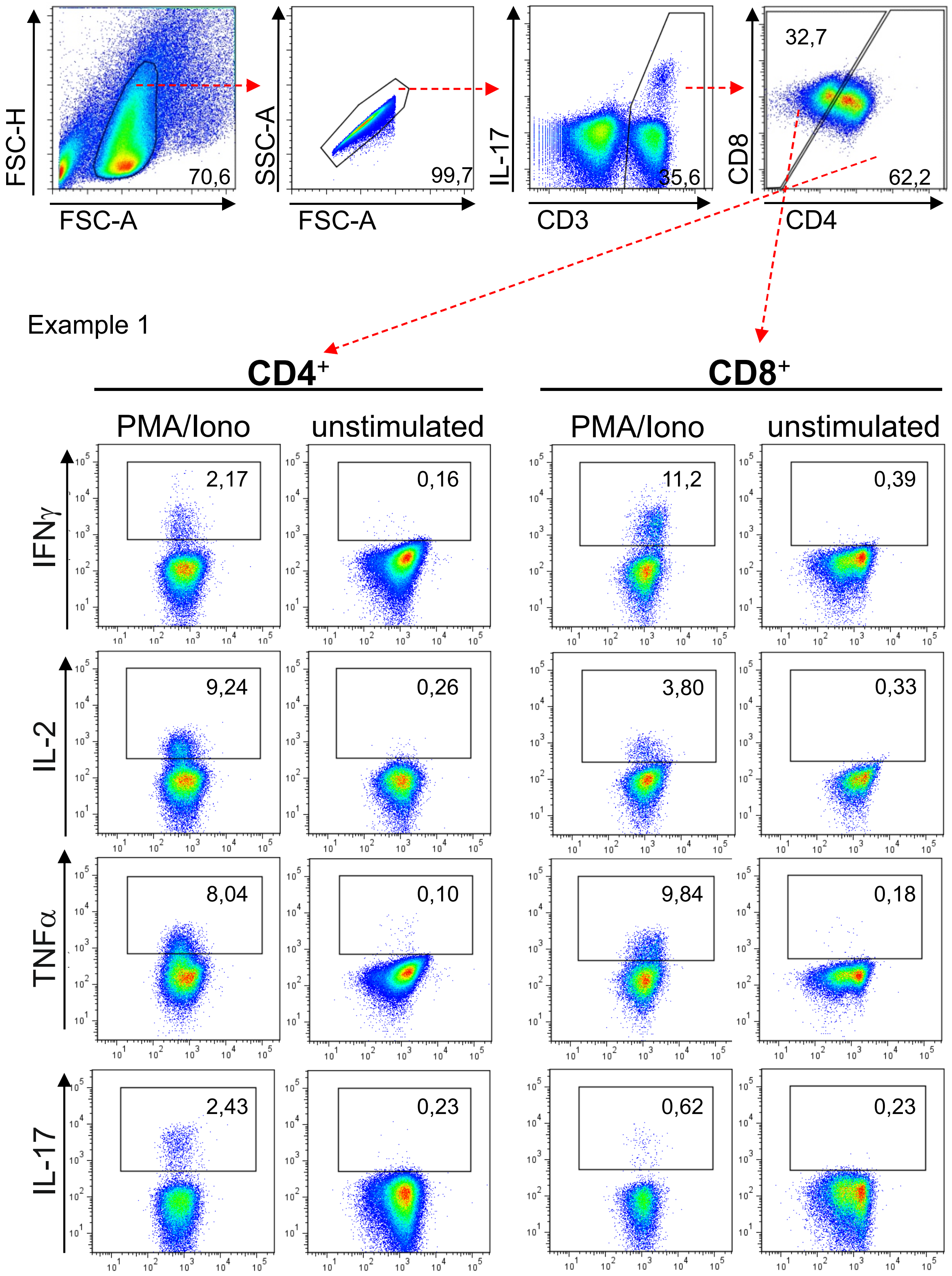

Supplemental Information

Figure S1

Example 2

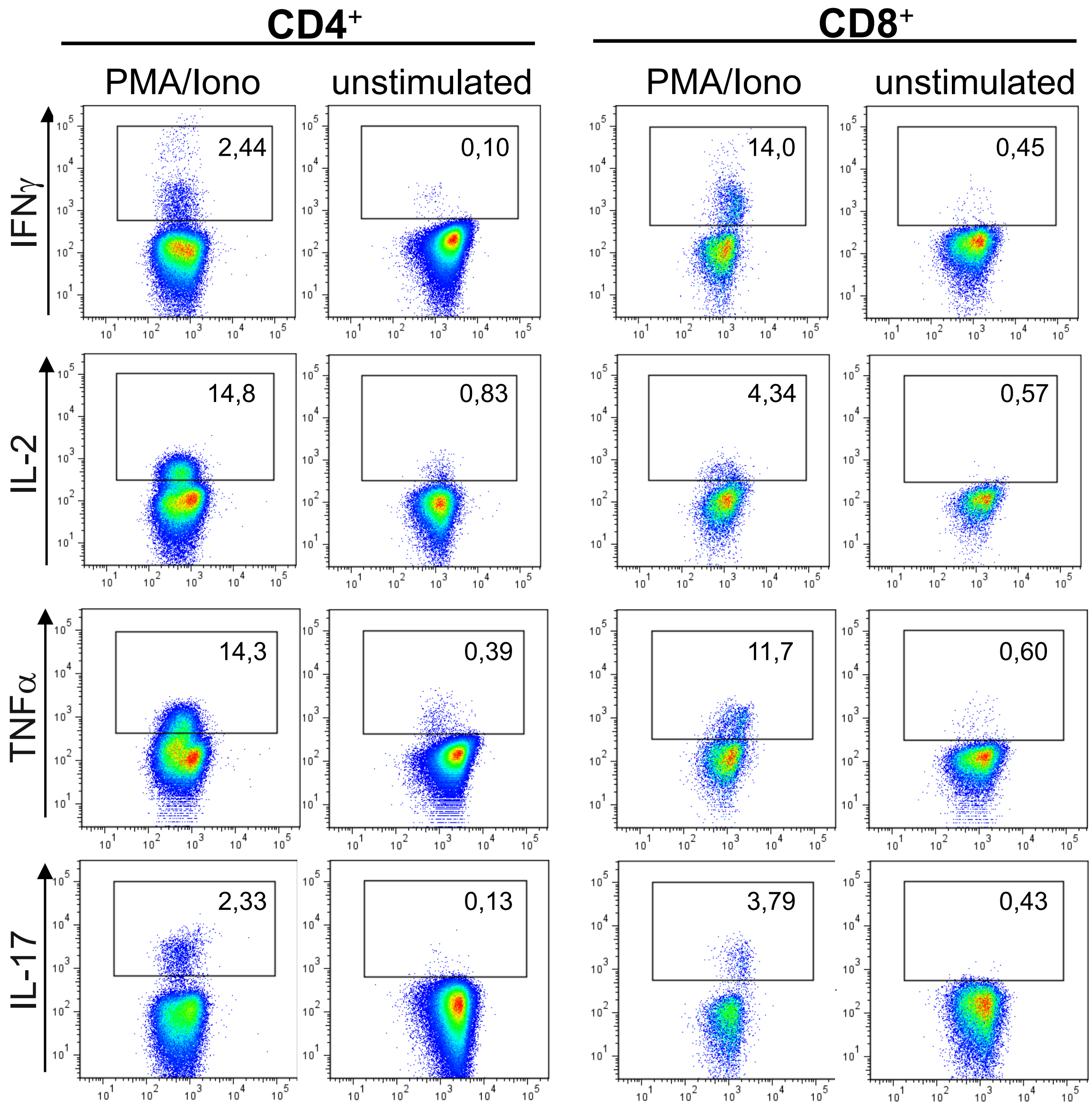

Figure S1: Gating strategy and controls for analysis of cytokin production via flow cytometry. Mononuclear cells (MC) were isolated from adenoids of children suffering from adenoid hypertrophy that underwent adenoidectomy. For analysis of cytokine expression cryopreserved MCs of children were left unstimulated (control) or stimulated with PMA and Ionomycin in the presence of Brefeldin A for 6 hr. Surface marker expression or cytokine production of $CD4^{+}$ and $CD8^{+}$ T-cells was determined by using flow cytometry. First intact MCs (SSC-A vs. FSC-A) and singlets (FSC-H vs. FSC-A) were gated followed by analyzing CD3 (Percp) and IL-17 (PE) expression to remove auto-fluorescence in some samples. CD4 (Pacific Blue) or CD8 (AmCyan) surface expression was determined from this gated population. Gates for intracellular cytokine expression (IFNg PECy7, IL-2 FITC, TNFa Cy5, and IL-17 PE) in PMA/Iono stimulated T-cells were drawn according to the unstimulated controls. Shown are cytokine frequencies of two representative examples.

Supplemental Information

Figure S2

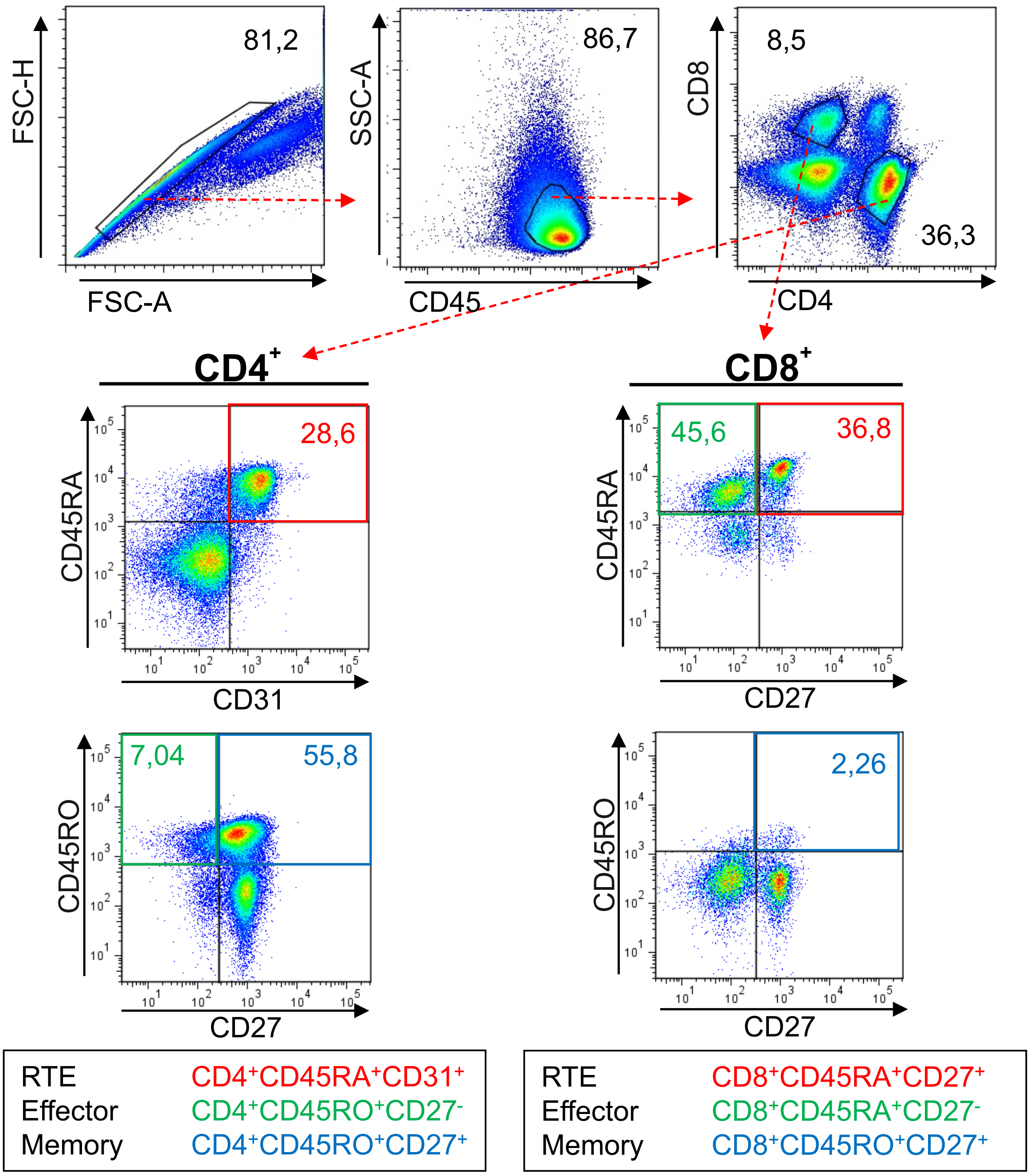

Figure S2: Gating strategy for phenotyping of CD4$^+$ and CD8$^+$ T-cells via flow cytometry. Mononuclear cells (MC) were isolated from adenoids of children suffering from adenoid hypertrophy that underwent adenoidectomy. For phenotyping of CD4+ and CD8+ T-cells cryopreserved MCs of children were left unstimulated and surface marker expression was determined to analyze by using flow cytometry. First singlets (FSC-H vs. FSC-A) and intact MCs (SSC-A vs. CD45 APC-Cy7) were gated followed by CD4 (Pacific Blue) and CD8 (AmCyan) expression. From this populations recent thymic emigrants (RTE), effector and memory T-cells were gated according to the surface markers shown in the boxes. Frequencies of one representative example is shown.
